# System identification of spiking neuron networks: a model-driven approach

**DOI:** 10.1186/1471-2202-12-S1-P215

**Published:** 2011-07-18

**Authors:** Daniele Linaro, Marco Storace, Maurizio Mattia

**Affiliations:** 1 Department of Biophysical and Electronic Engineering, University of Genoa, Genoa, Italy; 2 Department of Technologies and Health, Istituto Superiore di Sanità, Rome, Italy

## 

Understanding computational capabilities of the nervous system means to “identify” its emergent multiscale dynamics, possibly starting from the properties of its building blocks and following a “bottom-up” approach.

Here, we pursue this objective by adopting a model-driven identification, which we test on sparsely connected populations of excitatory integrate-and-fire (IF) neurons. Model neurons incorporate a fatigue mechanism underlying spike frequency adaptation (SFA) to lower discharge rates following a transient depolarization of the cell membrane potential [1]. We inspect the spontaneous relaxation time course of the population discharge rate without resorting to any linearization. The nonlinearity of the activity dynamics is exploited by aspecific supra-threshold stimulations in order to have a complete description of the system. This approach differs from those routinely employed in the study of the linear response properties of biological systems [2]. Furthermore, the network is investigated as a whole, the opposite of a bottom-up approach characterizing the system starting from its microscopic elements. Such description depicts the network dynamics in the low dimensional state space of the instantaneous discharge rate and the fatigue level of the adaptation mechanism, as suggested by a recent mean-field theory development [3].

Namely, from the elicited transient responses we work out the vector field of the reduced dynamics, and, based on the adopted theoretical framework, we extract the input-output gain function of the neurons in the network. The method allows testing the existence of an activity-dependent relaxation timescale for the network dynamics as expected from recent theoretical developments, extending the standard rate model framework *a la* Wilson and Cowan [4]. Besides, our model-driven identification makes available direct links to the microscopic level: indeed, we show how the decay time constant of the SFA, together with the absolute refractory period and the average synaptic efficacy, can be faithfully extracted. The robustness and generality of the introduced methodology is tested on relatively simple and well controlled *in silico* experiments, reporting a good agreement between theoretically expected and identified dynamics, even when phase transitions occur.

In conclusion, the devised identification has to be intended as a “middle-out” approach: starting from a mesoscopic description of the network dynamics, it provides in a “top-down” manner details about the microscopic domain at the cellular level. At the same time, the rate dynamics of a neuronal pool is quantitatively estimated, allowing for a “bottom-up” effective description of multi-modular macroscopic networks of neurons. The assumptions behind the underlying theoretical framework make the method of wide applicability to controlled biological preparations like cultured neuron networks and *in vitro* brain slices.
